# Quantification of Monosaccharides in Blood and Urine by HPAEC-PAD

**DOI:** 10.1021/acs.jafc.5c17160

**Published:** 2026-03-14

**Authors:** Paula Klügel, Lisa Seifert, Thomas Henle

**Affiliations:** Chair of Food Chemistry, 9169 Technische Universität Dresden, Dresden D-01062, Germany

**Keywords:** glucose, tagatose, fructose, galactose, HPAEC-PAD, metabolism, excretion, blood, urine

## Abstract

This study presents an analytical method for the quantification of glucose, fructose, tagatose, and galactose in plasma, erythrocytes, and urine using high-performance anion exchange chromatography with pulsed amperometric detection (HPAEC-PAD). The method has been validated with regard to linearity, LOD, LOQ, precision, and recovery over a wide concentration range. In oral glucose, fructose, and tagatose (containing galactose) tolerance tests. The analytical method proved to be suitable and reliable to examine the absorption and excretion of dietary monosaccharides. Postprandial plasma and erythrocyte levels of the four monosaccharides increased within 60 min and decreased to basal levels after 180 min, except for the plasma tagatose levels, which remained elevated. To our knowledge, this is the first time that an uptake of tagatose from plasma into erythrocytes has been shown. The renal excretion of ingested tagatose was only 3.7% within 24 h, while an oral glucose and fructose tolerance test had no influence on the renal excretion of dietary monosaccharides.

## Graphical abstract

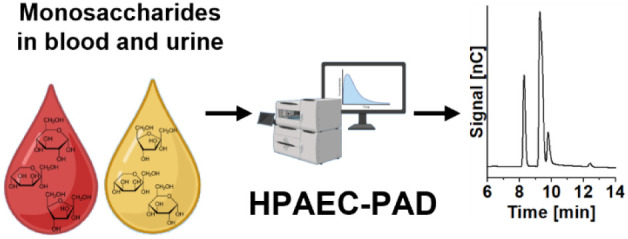

## Introduction

Hyperglycemia as it occurs in diabetes mellitus, can lead to cardiovascular diseases, nephropathy, neuropathy, retinopathy, and other serious health complications.^1^ Thus, good glycemic control and monitoring of blood glucose levels are crucial for reducing the risks of diabetic complications.^2^ Linked to that is the knowledge patients have about the glycemic indices (GI) of carbohydrate-rich foods.^3^ For both glycemic control and the calculation of GI, glucose analysis in physiological samples is essential and well established. But not only the quantification of glucose is of interest in physiological samples. Sugar substitutes, such as tagatose, a C4-epimer of fructose, are discussed as alternatives for glucose, fructose, or sucrose. Tagatose causes no changes in postprandial glucose levels in plasma and serum in healthy subjects and those with type 2 diabetes,^4–6^ leading to a decrease in HbA_1c_ levels in type 2 diabetes.^6,7^ However, the metabolic fate of tagatose remains only assumed based on its structural similarity to fructose. Thus, further absorption, distribution, metabolism, and excretion (ADME) studies investigating the potential of tagatose and other sugar substitutes as alternative sweeteners also require reliable sugar analysis in physiological samples such as plasma, erythrocytes, and urine.

There are several methods for quantifying sugars in physiological samples. Enzymatic methods are the most common for glucose in serum or plasma, where hexokinase (EC 2.7.1.1) or glucose oxidase (EC 1.1.3.4) are used.^4,5,8–12^ Enzymatic methods are also used for quantifying fructose and galactose in physiological samples.^13–16^ By combining suitable enzymes, it is possible to quantify two different sugars side by side.^13^ The strengths of enzymatic methods lie in their specificity and sensitivity, which allow more complex samples to be analyzed. However, these methods are limited by the availability of suitable enzymes and also have limited capabilities when it comes to simultaneously quantifying more than two sugars side by side.

Gas chromatography offers sensitivity and high resolution, especially when it is paired with mass spectrometry (GC-MS). It allows the profiling and quantification of sugars and sugar alcohols simultaneously. However, samples require derivatization, for example, oximation and silylation,^17,18^ to convert sugars into volatile and stable derivatives,^19^ as well as deproteinization and defatting.^20–22^ Tagatose can also be quantified by GC-MS.^20,23–26^ However, quantifying different monosaccharides simultaneously in physiological samples by GC-MS can be challenging because of the high amount of glucose contained. Glucose shows only small differences in retention time with derivatized compounds of similar molecular weight, such as other hexoses like fructose, galactose, or tagatose. This results in peak overlapping or coelution. The mass spectra for those samples are very similar, and only small differences occur, mainly related to fragment intensity.^25,26^

Another approach for sugar analysis is high-performance anion exchange chromatography with pulsed amperometric detection (HPAEC-PAD). Here, the weak acidity of the sugars is exploited, which is why they are deprotonated at high pH values. The advantage of this technique is its high sensitivity, the simultaneous analysis of different carbohydrate classes (mono-, di-, oligo-, polysaccharides, and sugar alcohols), the relatively uncomplicated sample preparation as there is no derivatization needed,^27^ and a relatively selective detection of carbohydrates due to the conditions used for PAD detection.^28^ At present, only a few studies have been published concerning the use of HPAEC-PAD for sugar analysis in physiological samples, mainly quantifying mannitol, rhamnose, xylose, 3-*O*-methylglucose, and lactulose in urine and plasma, as they are used in noninvasive investigations of active and passive intestinal mucosal transport.^29–34^ Application notes by a company manufacturing HPAEC equipment described methods to analyze galactose, glucose, sucrose, ribose, and lactose in synthetic urine.^35,36^ Seo et al. developed a method to quantify galactose and glucose in serum as diagnostic markers of diabetes and galactosemia.^37^ A more general approach was described by Feil and Lunn, who developed a protocol for analyzing various soluble sugars and sugar alcohols by HPAEC-MS/MS.^38^ However, sample preparation in this protocol is a multistep method, where a relatively high amount of dry sample is needed, which is not always possible for physiological samples.

Hence, the aim of this study was to develop a method for the quantification of glucose and fructose and—for the first time—for galactose and tagatose in physiological samples such as urine, plasma, and erythrocytes by HPAEC-PAD. This method can be used, for example, in studies on glycemic control, GI measurement, or in ADME studies of sugar substitutes.

## Materials and Methods

### Chemicals

The following substances were purchased from commercial suppliers: Ammonium chloride (p.a.), Creatinine (≥99%), Sodium citrate dihydrate (≥99%) (Merck, Darmstadt, Germany), Calcium chloride (water-free), Disodium hydrogen phosphate dihydrate (99%), Magnesium sulfate (99%), Potassium chloride (99.5%, p.a.), Sodium dihydrogen phosphate dihydrate (99%), Urea (99.5%) (Grüssing, Filsum, Germany), d­(−)-Fructose (≥99.5%), d­(+)-Glucose (p.a., ACS, water-free) (Carl Roth, Karlsruhe, Germany), d­(+)-Galactose (98%) (Alfa Aesar, Karlsruhe, Germany), Potassium oxalate monohydrate (≥99%), Uric acid (≥99%) (Sigma, Steinheim, Germany), d-Tagatose (99%), Trichloroacetic acid (99+%, ACS) (Thermo Scientific, Acros Organics, Geel, Belgium), Sodium chloride (≥99.5%), Sodium hydroxide solution (50% (w/w), extra pure), Sodium sulfate anhydrous (≥99%) (Fisher Scientific, Acros Organics, Geel, Belgium), Sodium acetate anhydrous (electrochemical grade) (Thermo Fisher Scientific, Sunnyvale, CA). Ultrapure water with a resistivity of 18.2 MΩ·cm and a total organic carbon (TOC) content of <5 ppb (ASTM Type I) was used for the preparation of all standard solutions, all samples, and the HPAEC-PAD eluents; it was obtained from an ELGA Labwater PureLab Classic UVF MK2 water purification system (London, UK). Artificial urine was prepared according to the literature.^39^

### Blood and Urine Samples

Blood and 24 h urine samples were collected from a healthy volunteer (female, age: 22 years, body mass index: 22.2 kg/m^2^). The volunteer underwent an oral glucose tolerance test (oGTT), an oral fructose tolerance test (oFTT), and an oral tagatose tolerance test (oTTT), where after an overnight fasting period (12 h), venous blood was obtained prior to and at 30, 60, 90, 120, and 180 min after a sugar load. For the oGTT, the volunteer consumed 75 g of glucose (Dextrose, K Classic, Bad Wimpfen, Germany) in 200 mL of water. For the oFTT, 40 g of fructose (Sucofin, TSI Consumer Goods, Zeven, Germany) in 200 mL of water was consumed. For the oTTT, the volunteer consumed 50 g of a tagatose-galactose mix (59.3% d-Tagatose, 40.5% d-Galactose, ≤0.2% Lactose, NuPrevento, Frankfurt (Oder), Germany). The blood was collected by using lithium heparin monovettes (Sarstedt, Hildesheim, Germany). The 24 h urine was collected on the days of the oral tolerance tests (09:00 a.m. until 09:00 a.m. the following day), with the first morning urine discarded. Baseline urine was collected in the same way on a different day with no oral tolerance test and an unrestricted diet.

The study has been approved by the Ethics Committee of Technische Universität Dresden (reference: SR-EK-18012020). Written consent was obtained from the volunteer.

### Blood Sample Preparation

After blood sampling, plasma and erythrocytes were separated by centrifugation (2000 *g*, 10 min, 4 °C). The buffy coat was discarded, and plasma was stored at −80 °C until analysis. Erythrocytes were washed three times with equal amounts of Krebs–Ringer phosphate buffer (135 mM NaCl, 5 mM KCl, 1.3 mM CaCl_2_, 1.2 mM MgSO_4_, 10 mM NaH_2_PO_4_, 5 mM glucose, pH 7.4), which was discarded after centrifugation (2000 *g*, 10 min, 4 °C). Erythrocytes were lysed by adding a 4-fold amount of water, mixing via vortex at 1000 rpm for 10 s, storing on ice for 10 min, and centrifugation (9500 *g*, 10 min, 4 °C). Lysed erythrocytes were stored at −80 °C. For analysis of the monosaccharides, the samples were thawed and centrifuged (9500 *g*, 10 min, 4 °C). 40 μL of plasma or lysed erythrocytes were added to 80 μL of trichloroacetic acid (TCA; 5%, w/v) and were mixed immediately for protein precipitation. After 10 min on ice, samples were centrifuged (9500 *g*, 10 min, 4 °C), and 40 μL of the supernatant were diluted with 365 μL of water. Modifications had been made for 0 and 180 min erythrocyte samples of the oFTT; 40 μL of the supernatant was diluted with 160 μL of water. For the modification of erythrocyte samples of the oTTT, all volumes of the samples and the reagents used for protein precipitation were doubled to be able to quantify tagatose. After protein precipitation, the sample preparation was the same as for the other samples. Samples were mixed well and transferred into HPAEC vials (0.3 mL, polypropylene, Macherey-Nagel, Düren, Germany) via a syringe filter (nylon, 0.2 μm,13 mm, Wicom, Heppenheim, Germany).

### Urine Sample Preparation

After sample collection, the 24 h urine sample was weighed to determine the urine volume. The required density of 24 h urine is, on average, 1.01 g/mL.^40^ Two aliquots of 15 mL were stored at −20 °C until analysis. After thawing and mixing well, 10 μL of urine was added to 1990 μL of water. Samples were mixed and transferred into HPAEC vials (0.3 mL, polypropylene) via a syringe filter (nylon, 0.2 μm, 13 mm).

### Calibration Preparation for Blood Samples

Quantification was performed by external calibration with the respective standards. The quantification of the monosaccharides in plasma and erythrocytes was conducted using mixtures of the reference standards in TCA ( 0.33% w/v end concentration) in the ranges of 0.1–200 μmol/L for tagatose and galactose, 5–400 μmol/L for glucose, and 0.1–40 μmol/L for fructose. The stability of the standards in TCA was tested, as they remained in the autosampler for some time. No changes in the concentration were observed.

### Calibration Preparation for Urine Samples

For the quantification of monosaccharides in urine, mixtures of the reference standards in artificial urine were prepared. Stock solutions of the respective standards were diluted in water, and 10 μL of artificial urine, according to the literature,^39^ were added, resulting in reference standards in the ranges of 0.1–10 μmol/L for glucose and fructose, 0.07–100 μmol/L for galactose, and 0.7–100 μmol/L for tagatose.

### Method Validation for Blood

The following parameters have been used for method validation: linearity, limits of detection (LOD) and quantification (LOQ), method precision, measurement precision, and recovery in high-concentration and low-concentration ranges.

The linearity was evaluated using Mandel’s fitting test according to the literature.^41^ The test variable (TV) is calculated according to eq 1 and compared with the corresponding *F*-value with 1 and *N* – 3 degrees of freedom at a significance level of *α* = 0.05 (*F*_0.05,1,*N* – 3_).

1 $$\mathrm{TV}=\frac{\left(N-2\right)\cdot s_{y1}^{2}-\left(N-3\right)\cdot s_{y2}^{2}}{s_{y2}^{2}}$$

The residual standard deviations of linear and quadratic regressions (*s*_*y*1_ and *s*_*y*2_) are calculated according to eqs 2 and 3.

2 $$s_{y1}=\sqrt{\frac{\overset{n}{\underset{i=1}{\sum }}{\left(y_{i}-{ŷ}_{i}\right)}^{2}}{n-2}},{ŷ}_{i}=a+b\cdot x$$

3 $$s_{y2}=\sqrt{\frac{\overset{n}{\underset{i=1}{\sum }}{\left(y_{i}-{ŷ}_{i}\right)}^{2}}{n-3}},{ŷ}_{i}=a+b\cdot x+c\cdot x^{2}$$

The null hypothesis H_0_ (no significant difference exists between the residual values) was rejected if TV > *F*_0.05,1,*N*__– 3_. As shown in Table 1, a quadratic regression model was more suitable for describing the correlation between signal and concentration for all analytes in erythrocytes and plasma, and for galactose, glucose, and fructose in urine, as TV was >*F*_0.05,1,*N* – 3_. For tagatose in urine, the null hypothesis cannot be rejected, as TV is < *F*_0.05,1,*N* – 3_, meaning there is no significant difference between the residual values of the linear and the quadratic regression model. In a case like this, the coefficients of determination (*R* ^2^) can be compared to decide which model fits best.^41^

**Table 1:** Range and Fit to a Regression Model (Mandel’s Test) of Galactose, Glucose, Tagatose, and Fructose in Erythrocytes, Plasma, and Urine

Fit regression model							
	Range [μmol/L]	*R* ^2^_linear_ ^a^	*R* ^2^_quadratic_ ^a^	*s*_*y*1_ ^b^	*s*_*y*2_ ^b^	*F* ^c^	TV^d^
erythrocytes and plasma							
galactose	0.5–200	0.9594	0.9978	1.8469	0.4816	6.608	83.2246
glucose	5–400	0.9875	0.9929	4.4828	1.9843	5.318	37.9323
tagatose	0.5–200	0.9784	0.9999	0.7148	0.0664	6.608	690.0950
fructose	0.1–40	0.9986	0.9998	0.0242	0.0111	7.709	19.7787
urine							
galactose	0.07–100	0.9788	0.9998	1.0933	0.4371	5.591	43.0522
glucose	0.1–25	0.9752	0.9981	0.5291	0.1609	6.608	59.8970
tagatose	0.7–100	0.9963	0.9982	0.2432	0.1952	7.709	3.6061
fructose	0.1–25	0.9826	0.9977	0.1524	0.0607	6.608	32.9057

a *R* ^2^, coefficient of determination.

b *s*_*y*1_ and *s*_*y*_2, residual standard deviations from the linear and quadratic regressions.

c *F*-value at α = 0.05 for 1 and *N* – 3 (*N*, number of measurements) degrees of freedom.

d TV, test variable by means of Mandel’s test. The TV is compared to *F* to reject or accept the H_0_ (no significant difference between the residual variances of the linear and quadratic regressions).

LOD and LOQ were determined with signal-to-noise ratios (S/N) of 3 and 10, respectively, from the analytes in TCA (0.33% (w/v)). A sample of fasting erythrocytes and fasting plasma was prepared and measured six times independently of each other for the method precision of glucose. Because galactose, fructose, and tagatose levels in fasting blood samples were <LOD, a mixture of standard references in TCA (0.33% (w/v)) with concentrations of 10 μmol/L for galactose and tagatose and 1 μmol/L for fructose was used for method precision. For the measurement precision of glucose, a sample of fasting erythrocytes and a sample of fasting plasma were prepared once and measured six times. For galactose, fructose, and tagatose, a mixture of standard references in TCA (0.33% (w/v)) with concentrations of 10 μmol/L for galactose and tagatose and 1 μmol/L for fructose was used. Recovery in high and low concentration ranges was determined according to literature.^41^

### Method Validation for Urine

The same validation parameters as for blood have been used. LOD and LOQ were determined with S/N of 3 and 10, respectively, from the analytes in artificial urine. For method precision, a mixture of standard references in artificial urine was prepared and measured six times independently of each other in the same way as for the samples. The concentration for galactose and tagatose was 10 μmol/L, and for glucose and fructose, 1.25 μmol/L. A standard mix of 1 μmol/L glucose and fructose and 10 μmol/L galactose and tagatose in artificial urine was analyzed six times to determine measurement precision. Recovery in high and low concentration ranges was determined according to literature.^41^

### HPAEC-PAD

The quantification of the monosaccharides was performed by HPAEC-PAD using a Dionex ICS-6000 system (Sunnyvale, CA), equipped with a Single Pump, an AS-AP Autosampler, a 10 μL injection loop (full loop injection used), and an Electrochemical Detector cell with a gold working electrode and an Ag/AgCl reference electrode. The carbohydrate 4-potential waveform was applied. The pulsed potential starts with a 0.2 s period that allows the charging current to decay at +0.1 V, followed by a 0.2 s detection period in which the current from analyte oxidation at +0.1 V is measured. A reductive cleaning period follows at −2.0 V for 0.01 s, activation and further cleaning at +0.6 V (0.01 s), and reduction at −0.1 V for 0.06 s.

The eluents A and B were 0.9 mmol/L NaOAc + 4.2 mmol/L NaOH (A) and 250 mmol/L NaOH (B). The eluents were kept under an N_2_ atmosphere using a direct connection to an EO gas regulator (constant pressure of 3–6 psi).

The SweetSep AEX20 column (5 μm, 4 mm × 200 mm, Antec Scientific, Alphen aan den Rijn, The Netherlands), with the corresponding guard column, was used.

Analysis was conducted at 10 °C autosampler temperature and 30 °C column temperature using a flow rate of 0.6 mL/min with the starting conditions of 100% A. This solvent composition was held for 15 min, then changed to 100% B within 0.2 min, and held at 100% B for 30 min for analysis of the blood samples and for 15 min for the urine samples as a washing step. It changed to 100% A within 0.2 min and was finally held at 100% A for 30 min for column equilibration.

### Statistical Analysis

Data are shown as the mean of a dual determination with the corresponding standard deviation. The investigation of the suitability of linear and quadratic regression as calibration models, applying Mandel’s fitting, and the statistical evaluation of the method validation experiments were carried out with MS Excel 2010 and OriginPro 2021b.

## Results and Discussion

### Identification of the Monosaccharides

The aim of this study was to develop a method for the simultaneous quantification of different monosaccharides in physiological samples. The chromatograms of the monosaccharides in plasma, erythrocytes, and urine are shown in Figure 1 with the results obtained in the oTTT and oFTT, and in Figure 2 with the results of the oTTT as examples. All compounds in the HPAEC-PAD profiles were identified by comparing the retention times with reference standards. The standards were analyzed in artificial urine and in TCA (0.33% (w/v), end concentration) individually, as well as in a standard mix. The chromatogram of all standards showed only one peak when analyzed individually, indicating no isomerization of one sugar to another during analysis (data not shown).

**Figure 1:**
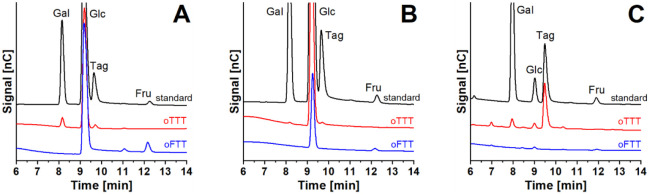
Chromatograms of galactose, glucose, fructose, and tagatose following an oTTT and an oFTT: (A) plasma, (B) erythrocytes, (C) urine by HPAEC-PAD. Gal: galactose, Glc: glucose, Tag: tagatose, Fru: fructose.

**Figure 2:**
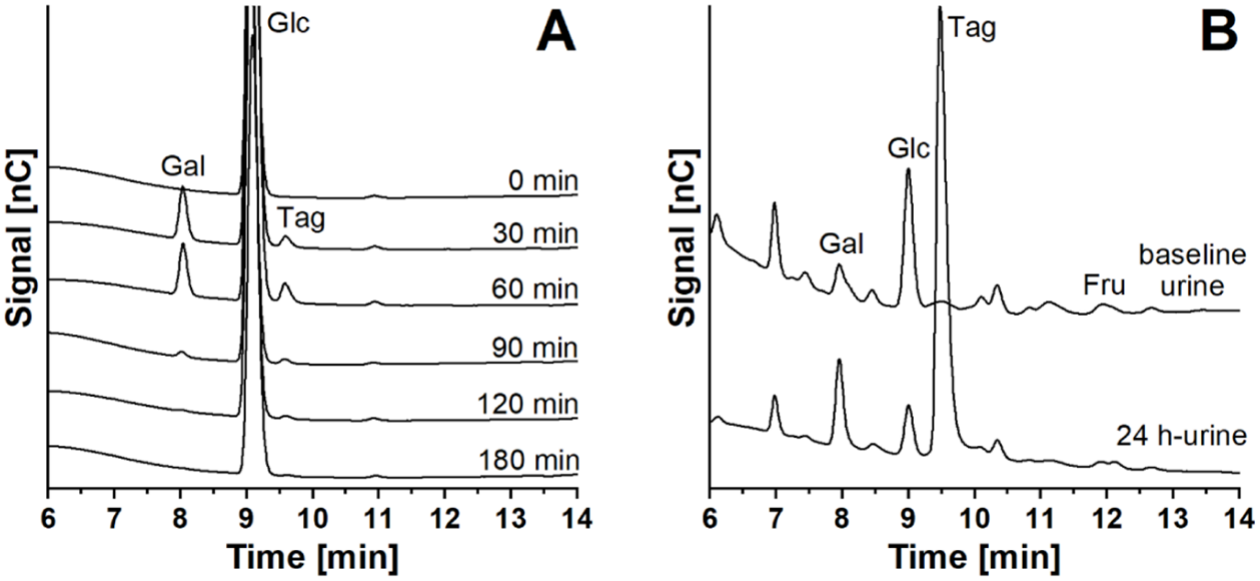
HPAEC-PAD chromatogram of galactose, glucose, and tagatose in plasma (A) and in urine (B) of a healthy participant following an oTTT. Gal: galactose, Glc: glucose, Tag: tagatose, Fru: fructose.

### Validity of the Method

The method presented was checked and validated regarding its linearity, LOD, LOQ, method precision, measurement precision, and recovery in high and low concentration ranges, according to literature.^41^

According to existing HPAEC-PAD methods for the quantification of monosaccharides, a quadratic function was suitable as a calibration model (see Materials and Methods and Table 1).^42,43^ For tagatose in urine, no significant difference between the quadratic and the linear regression model occurred, based on Mandel’s fitting test (see Table 1). *R* ^2^ of the quadratic regression model was closer to 1 when compared with the linear regression model (0.9982 vs 0.9963). Therefore, tagatose in urine was also analyzed based on a quadratic regression model, which simplifies the analysis in the different sample matrices, as all analytes could be evaluated using the same regression model.

In Table 2, all validation parameters for the monosaccharides analyzed in erythrocytes, plasma, and urine are shown. The LOD and LOQ were in the ranges of 0.1–5.3 μmol/L and 0.4–15.9 μmol/L, respectively. The monosaccharides added to the three physiological matrices were recovered at 88.8–101.3% in erythrocytes, at 81.8–101.6% in plasma, and at 93.5–128.5% in urine in a low concentration range. For a high concentration range, the recovery was 94.4–105.1% in erythrocytes, 72.6–109.0% in plasma, and 88.1–94.1% in urine, respectively. This indicates good selectivity and accuracy of the method, which is comparable to the literature.^36,37^ The method precision and the measurement precision varied between 1.3–11.4% and 0.3–6.2%, respectively. Biological samples are not as standardizable as food samples or other samples. Therefore, the acceptance criteria for precision of biological matrices go up to 15%,^44^ which indicates acceptable precision for all analytes in the three matrices analyzed.

**Table 2:** LOD, LOQ, Method and Measurement Precision and Recovery of Galactose, Glucose, Tagatose, and Fructose in Erythrocytes, Plasma, and Urine

	LOD [μmol/L]	LOQ [μmol/L]	Method precision [%]	Measurement precision [%]	Recovery [%] low concentration	Recovery [%] high concentration
erythrocytes						
galactose	0.9	2.7	1.8	1.6	101.3^a^	102.7^f^
glucose	0.7	2.0	1.3	1.1	98.3^b^	94.4^g^
tagatose	2.5	7.6	6.0	6.2	95.5^a^	105.1^f^
fructose	5.3	15.9	1.7	3.7	88.8^c^	101.7^h^
plasma						
galactose	0.2	0.5	1.8	1.6	101.6^a^	102.3^f^
glucose	0.1	0.4	4.8	0.3	81.8^b^	72.6^g^
tagatose	0.5	1.5	6.0	6.2	93.9^a^	109.0^f^
fructose	1.0	3.1	1.7	3.7	92.7^c^	107.9^h^
urine						
galactose	1.4	4.4	9.3	0.9	99.9^d^	93.3^i^
glucose	0.4	4.0	10.5	0.9	128.5^e^	94.2^j^
tagatose	3.5	11.5	8.7	1.2	95.5^d^	88.1^i^
fructose	4.0	13.5	11.4	1.2	93.5^e^	91.0^j^

a *c* = 5 μmol/L.

b *c* = 150 μmol/L.

c *c* = 0.5 μmol/L.

d *c* = 1 μmol/L.

e *c* = 0.25 μmol/L.

f *c* = 200 μmol/L.

g *c* = 250 μmol/L.

h *c* = 20 μmol/L.

i *c* = 75 μmol/L.

j *c* = 7.5 μmol/L.

### Plasma Levels of Glucose, Fructose, Tagatose, and Galactose

Using this method, galactose, glucose, tagatose, and fructose in erythrocytes, plasma, and 24 h urine of a healthy volunteer, who underwent an oGTT, oFTT, and oTTT, were analyzed. Data for plasma glucose following an oGTT are shown in Figure 3 A. Fasting plasma glucose was (5.3 ± 0.5) mmol/L, which increased to (7.3 ± 0.8) mmol/L within 30 min after ingestion of 75 g of glucose. The level decreased to the basal level after 120 min. This course of the curve following an oGTT was comparable with that reported for healthy participants in other studies,^4,9^ indicating the suitability of the presented method for glucose analysis in plasma.

**Figure 3:**
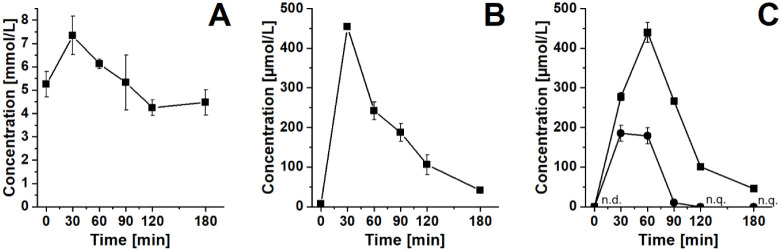
Plasma sugar levels of a healthy participant during an oral tolerance test. Glucose levels during an oGTT over time (A). Fructose levels during an oFTT over time (B). Tagatose (■) and Galactose (●) levels during an oTTT (C). n.d. < LOD (1.4 μmol/L), n.q. < LOQ (4.4 μmol/L). Data are shown as means ± SD.

The maximum plasma fructose levels following an oFTT were also reached 30 min after ingestion of 40 g of fructose ((454 ± 2) μmol/L, Figure 3 B). Teff et al. carried out a study in which 17 obese participants drank a fructose-sweetened beverage (15%, w/v) with every meal within 24 h. In this study, the plasma fructose levels increased up to 500 μmol/L as well, but within 60 min. They quantified plasma fructose levels using an enzymatic assay.^45^ UPLC-MS/MS was used in a study analyzing serum fructose after a 15 g fructose load. A maximum in serum fructose levels occurred at 60 min, but no blood was drawn at 30 min.^46^ In an animal study with rhesus macaques, the highest fructose levels were measured at 30 min.^15^

We were able to detect an increasing tagatose peak in the chromatogram of plasma samples following an oTTT with a tagatose load of approximately 30 g and a galactose load of approximately 20 g (Figure 2A). Plasma tagatose levels increased from <LOD to (439 ± 25) μmol/L within 60 min, which is comparable to the oFTT. Even after 180 min, plasma tagatose was still quantifiable (Figure 3 C). Plasma galactose levels increased from <LOD to (185 ± 20) μmol/L within 30 min and decreased to <LOQ within 120 min following an oTTT with a galactose load of approximately 20 g and a tagatose load of approximately 30 g. The tagatose-galactose load had no influence on plasma glucose and fructose levels (data not shown). Data on tagatose levels in blood are limited. To our knowledge, only two studies have quantified tagatose in serum and plasma up to now. In one of the studies, the serum concentration peaked after 50 min with a range of 50–280 μmol/L after a 30 g tagatose load, and only after 420 min could no tagatose be detected in serum in any subject.^5^ In the other study, the participants ingested 75 g tagatose 30 min prior to an oGTT. Plasma tagatose levels peaked at 90 min with a mean of (200 ± 39) μmol/L.^4^

### Erythrocyte Levels of Glucose, Fructose, Tagatose, and Galactose

Following an oGTT, the erythrocyte glucose levels increased slightly from (5.1 ± 0.5) to (5.6 ± 0.4) mmol/L within 60 min (Figure 4A).

**Figure 4:**
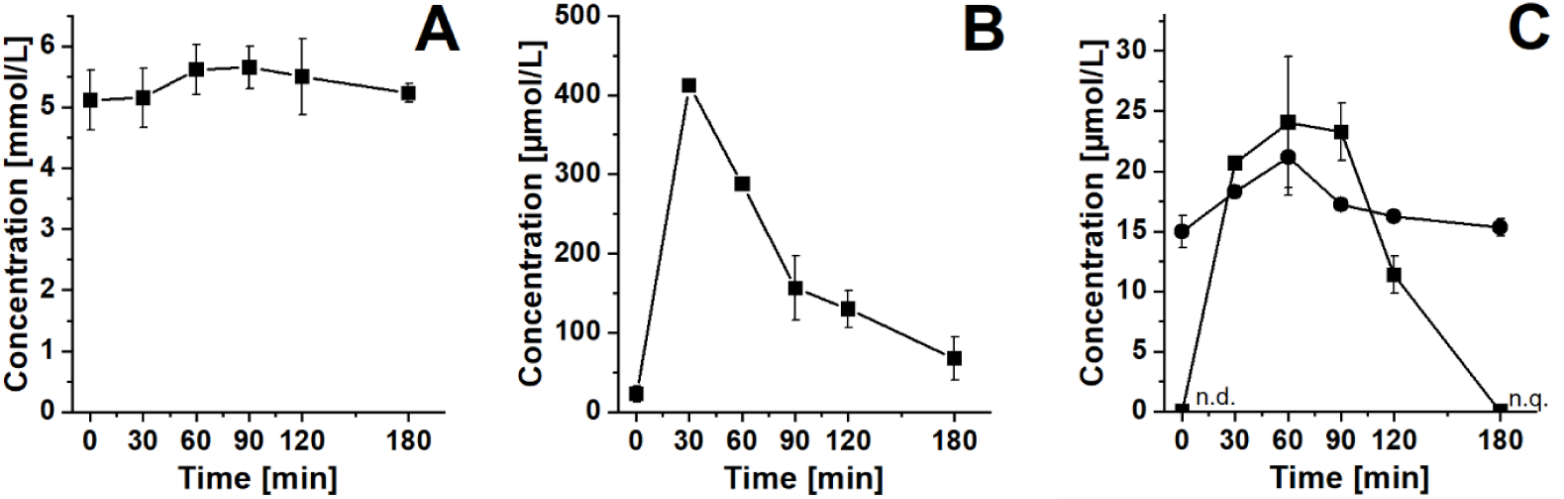
Sugar levels in erythrocytes of a healthy participant during an oral tolerance test. Glucose levels during an oGTT over time (A). Fructose levels during an oFTT over time (B). Tagatose (■) and galactose (●) levels during an oTTT (C). n.d. < LOD (2.5 μmol/L), n.q. < LOQ (7.6 μmol/L). Data are shown as means ± SD.

Fasting erythrocyte fructose levels were (23 ± 10) μmol/L. They increased to (412 ± 8) μmol/L within 30 min following an oFTT and decreased to (68 ± 27) μmol/L after 180 min (Figure 4 B). However, the fasting erythrocyte fructose levels were higher than those in another study ((9 ± 2) μmol/L, originally in nmol/g Hb, calculated with approximately 340 mg Hb/mL erythrocytes^47,48^ ), where fructose levels were analyzed using an enzymatic assay.^49^ The increase indicates an uptake of fructose into erythrocytes from plasma. GLUT5 has already been identified as the transporter for this uptake.^50,51^ The decrease in erythrocyte fructose levels indicates that erythrocytes are capable of metabolizing fructose. Two different pathways are known to date. Hexokinase phosphorylates fructose to fructose-6-phosphate, which is an intermediate of glycolysis. However, the affinity of hexokinase for fructose is several orders of magnitude lower than for glucose.^52–54^ Thus, this pathway does not explain the metabolization of fructose sufficiently. The second pathway is the phosphorylation to fructose-3-phosphate by a specific 3-phosphokinase,^55,56^ which could lead to the formation of 3-deoxyglucosone and protein glycation.

Data on postprandial erythrocyte tagatose and galactose levels following an oTTT with a tagatose load of approximately 30 g and a galactose load of approximately 20 g are shown in Figure 4C. Erythrocyte tagatose levels increased from <LOD to (24 ± 5) μmol/L within 60 min, showing an uptake of tagatose from plasma into erythrocytes after oral ingestion. After 180 min, the tagatose levels in erythrocytes decreased to <LOQ. The postprandial galactose levels in erythrocytes increased, with the maximum at 60 min, and decreased to basal levels at 180 min following an oTTT.

### Renal Excretion

The oGTT and oFTT showed no significant influence on the renal excretion of glucose and fructose (data not shown). However, for the oTTT, the galactose peak increased, and a new peak appeared in the chromatogram of 24 h urine when compared with the baseline urine, which could be identified as tagatose (Figure 2 B). In animal studies, the renal excretion of tagatose ranged between 5.5% (rat)^57^ and 3–7% (pig).^58,59^ In human studies, the range was even higher, with 0.7–5.3%,^5,8^ In the human studies, urinary tagatose was analyzed using a GC-MS method according to Jansen et al.^20^ In the present study, the volunteer ingested approximately 30 g of tagatose with the oTTT. (1100 ± 340) mg of tagatose was excreted with the 24 h urine, resulting in an excretion of (3.7 ± 1.1)% of the amount ingested, which indicates nearly complete metabolization of tagatose after absorption. Thus, we were able to reproduce the results of the human studies with our newly developed method using HPAEC-PAD. Even though galactose excretion increased, only 0.3% of the amount ingested was recovered in 24 h urine. That was expected, as galactose is mainly eliminated in the liver. Thus, no significant amounts are excreted in the urine.^60^

In conclusion, we developed a method for the simultaneous analysis of four different monosaccharides in blood and urine by HPAEC-PAD. We showed that a quadratic regression model is the most suitable one for the quantification and validated the method in accordance with recognized literature. The applicability of the method was proven for analyzing plasma, erythrocyte, and urine samples in an oGTT, as we were able to reproduce the typical postprandial course of plasma glucose levels following an oGTT in a healthy volunteer. Following an oGTT, oFTT, and oTTT, plasma and erythrocyte levels of fructose, tagatose, and galactose increased to a maximum after 30 or 60 min and decreased to basal levels in the oFTT and oTTT, indicating an uptake of the monosaccharides into erythrocytes from plasma and a potential metabolization in the cells. We showed a renal excretion of (3.7 ± 1.1) % tagatose within 24 h. We will apply this newly developed analysis method in further studies of the metabolism and excretion of tagatose and other sugars.

## Abbreviations Used

ADME: absorption, distribution, metabolism, excretion
Fru: fructose
Gal: galactose
GC-MS: gas chromatography coupled with mass spectrometry
GI: glycemic index
Glc: glucose
G-6-P: glucose-6-phosphate
HbA_1c_: glycated hemoglobin
HPAEC-MS: high-performance anion exchange chromatography coupled with tandem mass spectrometry
HPAEC-PAD: high-performance anion exchange chromatography coupled with a pulsed amperometric detector
LOD: limit of detection
LOQ: limit of quantification
NADP^+^/NADPH: nicotinamide adenine dinucleotide phosphate (oxidized and reduced form)
oGTT: oral glucose tolerance test
oFTT: oral fructose tolerance test
oTTT: oral tagatose tolerance test
S/N: signal-to-noise ratio
Tag: tagatose
TCA: trichloroacetic acid
TV: test variable
